# P53 expression is significantly correlated with high risk of malignancy and epithelioid differentiation in GISTs. An immunohistochemical study of 104 cases

**DOI:** 10.1186/1471-2407-8-204

**Published:** 2008-07-23

**Authors:** Ursula Pauser, Nina Schmedt auf der Günne, Günter Klöppel, Hartmut Merz, Alfred C Feller

**Affiliations:** 1 Department of Pathology, University of Lübeck, Germany; 2 Department of Pathology, University of Kiel, Germany; 3 Department of Gastroenterology, Albertinen Hospital Hamburg, Germany

## Abstract

**Background:**

Molecular analyses of the *c-kit *and *PDGFRα *genes have contributed greatly to our understanding of the development of gastrointestinal stromal tumors (GISTs), but little is known about their malignant potential. The aim of our study was to evaluate cell cycle regulators as potential prognostic markers in GISTs.

**Methods:**

We investigated 104 KIT positive GISTs from various tumor sites in immunoassays on CD34, Ki67 and particularly on P53, BCL-2 and Cyclin D1. The results were compared with tumor size, mitotic rate, proliferative activity, histological subtype, nuclear atypia and risk assessment according to Fletcher and Miettinen. Occurrence of metastases and survival were also taken into account.

**Results:**

The expression of P53 was significantly correlated with high risk criteria towards malignancy and epithelioid differentiation in GISTs. Likewise P53 label correlated significantly with the established prognostic indicators: tumor size, mitotic rate, nuclear atypia and proliferative activity. Regarding the site of tumor presentation, P53 was not a decisive factor. BCL-2 and Cyclin D1 expression was not related to any of the prognostic indicators.

**Conclusion:**

The present data identified P53 being a recommendable marker for predicting the risk of malignancy in GISTs. In addition, we found P53 significantly correlated with epithelioid tumor differentiation, independent of tumor site. BCL-2 and Cyclin D1, however, did not prove to be deciding markers for diagnosis and prognosis.

## Background

In recent years, progress has been achieved in establishing accurate diagnoses of gastrointestinal stromal tumors (GISTs), elucidating their molecular pathogenesis and developing a specific treatment. GISTs are defined as mesenchymal tumors of stomach and intestine that express KIT (CD117) and show either a *c-kit *mutation or a mutation in the gene that encodes the platelet-derived growth factor receptor alpha (PDGFRα) [1-3]. Mutations in both genes lead to ligand independent dimerization of transmembrane tyrosine kinase receptor type III and to intracytoplasmic signal transduction via phosphorylation of tyrosine kinase [4-7]. The tyrosine kinase receptor inhibitor imatinib (Glivec ^®^) has proven to be highly successful for the treatment of metastasized GISTs. Currently, (neo-) adjuvant imatinib therapy is being tested in order to minimize the risk of metastasis. The histological appearance of GISTs is variable, but in general there are two patterns: epithelioid and spindle cell [8,9]. Epithelioid GISTs have been described with a variable nest-like, trabecular and pleomorphic differentiation [9].

Tumor size and mitotic rate are proven criteria for predicting the risk of malignancy [10,11]. Tumor localization and histological differentiation correlate with prognosis, too [11-14]. GISTs in general have a high malignant potential, because up to 50% of patients suffer from metastases already at the time of primary diagnosis [9].

Criteria that predict malignant potential could help clinicians to treat only patients who need therapy. Such criteria should minimize side effects and costs for cases of low metastatic risk [12,13]. However, there is little information about the malignant development of GISTs. That risk may increase with the number of acquired genetic alterations in regulator genes, such as cellular oncogenes or suppressor genes [15].

The tumor suppressor P53 and the antiapoptotic protein BCL-2 attract particular attention, because they are involved in DNA repair and cell death. Functional loss of P53 promotes transformation, whereas BCL-2 blocks apoptosis and enhances proliferation. Several studies have addressed the impact of P53 on GIST prognosis using varying cut off levels from 1% to 50% [16-20]. BCL-2 expression has been extensively investigated in gastrointestinal mesenchymal tumors including GISTs, but results were heterogeneous [17,19,21-23].

Cyclin D1 promotes G1/S transition [24,25] by regulating the activity of the Cyclin-dependent protein kinases CDK4 and CDK6, and interferes with cell survival and division [26]. Cyclin D1 overexpression has been found implicated in several types of human neoplasias [27]. Reduced expression of Cyclin D1 and poorer outcome has been detected in breast carcinomas [28]. and in a few GISTs [29]. Others reported significant correlation between Cyclin D1 expression and outcome or histological pattern [20,30].

The aim of the present study was to evaluate cell cycle regulators as potential prognostic markers in GISTs. Beside KIT, CD34 and Ki67 antigen, we investigated the expression of P53, BCL-2, and Cyclin D1. The data from GISTs of the upper and lower gastrointestinal tract were correlated not only with established risk factors (tumor size, mitotic rate and proliferative activity), but also with cytological subtype, tumor site, occurrence of metastasis and overall survival. Thus, site-independent risk assessment according to Fletcher [10] and site-dependent criteria of malignancy according to Miettinen were considered [11,31] since these criteria are being used to decide on (neo-) adjuvant therapy.

## Methods

### Surgical specimens and histology

The pathology departments at the universities of Brussels (Belgium), Varese (Italy) and Kiel (Germany) provided a host of primary gastrointestinal mesenchymal tumors that had been formalin-fixed, paraffin embedded and collected during the period 1973–2001 [32]. None of the tumors was treated with imatinib. The retrospective study was undertaken in accordance with institutional guidelines. Informed consent for scientific evaluation had been obtained from patients at their primary clinical treatment.

Serial sections were made from the tissues mentioned above and stained with hematoxilin and eosin, then subject to a KIT immunoassay to identify GIST cases. We detected 104 KIT-positive tumors, 10 of which came from Brussels, 46 from Varese, and 48 from Kiel. The collective consisted of 59 female and 45 male patients; their ages ranged from 24 to 90 years (mean 62). The tumors had been resected from the upper and lower gastrointestinal tract (stomach 60, small intestine 37 and large intestine 7). The GIST sizes ranged from 0.4 cm to 30 cm in diameter (mean 5.5 cm); 42% were smaller than 5 cm (12% < 2 cm) and 58% ≥ 5 cm (21% < 10 cm).

Follow-up information was available on 49 patients. During a period of up to 284 months (mean 75), 33 patients survived disease free. Further 6 patients were still alive at the end of the follow-up, but sustained metastases in liver, omentum and/or peritoneum (5) or a local recurrence (1). Ten patients died of the disease, all of them had metastases. (Follow-up was not available for 5 patients who had metastases at the time of primary diagnosis.)

The tumor sections were reviewed and classified as epithelioid or spindle cell type according to the dominant pattern. Nest-like, trabecular and pleomorphic differentiation was taken as a predominantly epithelioid subtype [9]. Nuclear atypia was graded as being absent, mild or severe. The risk of malignancy was classified according to Fletcher's [10] and Miettinen's [11] criteria.

### Immunohistochemistry

Staining reactions for KIT, CD34, Ki67 antigen, P53, BCL-2 and Cyclin D1 were performed using commercially available antibodies (Table 1). Prior to immunostaining with the specific antibodies, the slides were pretreated with citric acid in a pressure cooker for antigen retrieval. Endogenous peroxidase was inactivated with H_2_O_2 _blocking reagent (DAKO Cytomation, Hamburg, Germany) for 20 min. Slides were incubated with the primary antibodies at room temperature for 30 min, then immunostained using the avidin-biotin complex peroxidase method (ABC Elite Kit, Vector Laboratories, Burlingham, CA). Complexes were visualized with diaminobenzidine (DAB substrate kit, Vector Laboratories).

**Table 1:** List of primary antibodies

**Antigen**	**Antibody**	**Dilution**	**Source**
BCL-2	124	1:100	DAKO Cytomation, Glostrup, Denmark
CD34	QBEND 10	1:1500	Immunotech, Marseille, France
Cyclin D1	P2D11F11	1:10	Novocastra Laboratories Ltd, Newcastle, UK
Ki67	Ki-S5	1:1000	Dept. Hematopathology, University of Kiel, Germany
KIT	K1906	1:30	DAKO Cytomation, Glostrup, Denmark
P53	DO-1	1:20	Calbiochem (Merck, Darmstadt, Germany)

Without knowledge of the clinical data, UP and NSG evaluated independently all tumor specimens by light microscopy. Most of the judgments agreed; doubtful cases were scrutinized once more or discarded.

Control tissues were added to every staining patch. The immunostaining of P53 was controlled particularly upon decorated nuclei in the mucous membrane, whereas normal stromal tissue, which does not proliferate, remained unstained (negative control). Therefore all GISTs, in which cells showed an intensive nuclear reactivity for P53, were considered positive. This 0% cut-off decision was further supported by the fact that the mutated P53 has a longer half-life than the wild type protein [33]. Also for Cyclin D1, all positive nuclear reactions were counted; however, staining artefacts reduced this sample to 80 specimens.

For KIT, CD34 and BCL-2, clear cytoplasmic labels were considered positive. Ki67 indicates proliferative activity during the entire cell cycle, except for the G0 phase. The percentage of Ki67 positive nuclei was reported from 1,000 tumor cells within representative areas.

### Statistical analysis

The correlation between P53, BCL-2 and Cyclin D1 expression as well as tumor size, mitotic rate, proliferative activity, nuclear atypia, occurrence of metastasis, tumor differentiation, tumor site and Fletcher's risk criteria was analyzed by means of the Pearson chi-square test and Fisher's exact test. The software package SPSS 13.0 for Windows (SPSS Inc, Chicago, IL) was used for these calculations. Statistical significance was assumed at p < 0.05. Overall survival was evaluated according to Kaplan-Meier (not shown).

## Results

The histological record yielded spindle cell differentiation with whorl-like or palisading patterns in 67 GISTs (64%). Pure or predominantly epithelioid differentiation with polygonal tumor cells containing clear cytoplasm was found in 37 GISTs (36%). The mitotic rate ranged from 0–125 nuclear divisions (mean 3.5; median 11) when counted for each patient within 50 microscopic high power fields (HPF). In detail, the mitotic rates were less than 5/50 HPF in 53 GISTs, 5–10/50 HPF (n = 15), and more than 10/50 HPF (n = 36). Nuclear atypia was mild in 39 cases and severe in 38, but 27 GISTs did not show any. According to Fletcher's criteria, the risk of malignancy appeared very low in 12 GISTs, low in 26, intermediate in 18 and high in 48 cases.

Metastases had developed only in GISTs with intermediate (3/3) or high (17/27) risk of malignancy (p = 0.001). Patients with metastases had a significantly poorer outcome than those without (p = 0.011). The proliferative activity, detected upon Ki67, ranged from 0–46% (median 7%). A threshold of 7% was applied for statistical analysis. Thus, minor proliferative activity allocated in 67 of 104 cases, while it was ≥ 7% in 37 GISTs.

**Figure 1:**
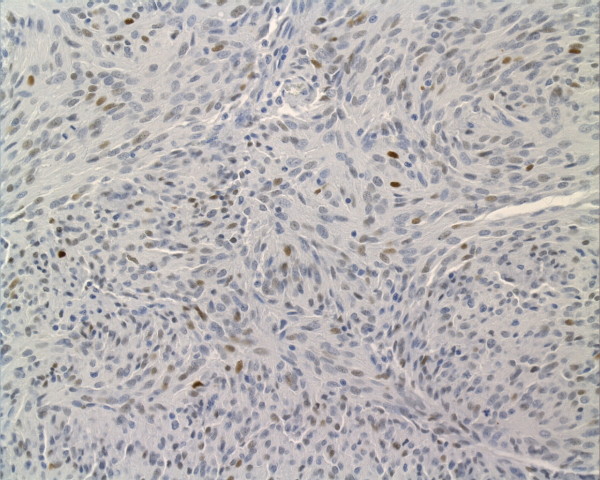
Nuclear P53 expression in a metastasized duodenal GIST, 8 cm in diameter, showing spindle cell differentiation.

Scattered, but strong nuclear P53 decoration characterized 38% of the tumors (40/104) (Fig. 1). The expression of P53 was significantly correlated with high mitotic rate (p = 0.013), high proliferative activity (p = 0.01), severe nuclear atypia (p = 0.02) and with the epithelioid subtype (p = 0.002). P53 was significantly correlated with Fletcher's criteria (p < 0.0001). P53 expression was detected most often in GISTs originating in the colon (71%), followed by GISTs deriving from the small intestine (46%) and stomach (32%); these observations, however, were statistically not significant (for details see Tables 2 and 3).

**Table 2:** P53, BCL-2 and Cyclin D1 expression in GISTs related to clinicopathological features

	Tumor size (cm)				Mitotic rate/50 HPF				Malignancy risk **F**			Malignancy risk **M**		
				
	<2	2–5	5–10	>10	<5	5–10	>10	very low	low	intermediate	high	prob benign	low	prob malign
**P53**	**2/10**	**7/31**	**22/37**	**9/22**	**13/50**	**7/15**	**20/35**	**1/10**	**3/25**	**10/18**	**26/47**	**3/24**	**5/17**	**32/59**
	**20%**	**23%**	**37%**	**41%**	**26%**	**47%**	**57%**	**10%**	**12%**	**56%**	**55%**	**13%**	**29%**	**54%**
BCL2	5/9	22/30	30/37	15/22	34/48	9/15	29/35	4/8	18/25	15/18	35/47	13/22	14/17	45/59
	56%	73%	81%	68%	71%	60%	83%	50%	72%	83%	74%	59%	82%	76%
CyD1	3/7	12/26	15/29	4/18	19/40	5/12	10/28	2/5	10/21	8/16	14/38	6/18	8/15	20/47
	43%	46%	52%	22%	48%	42%	36%	40%	47%	50%	37%	33%	53%	42%

	Nuclear atypia			Proliferative activity		Histological subtype		Tumor site						
					
	absent	mild	severe	<7%	≥ 7%	spindle cell	epithelioid	stomach	small intestine	large intestine				

**P53**	**6/26**	**13/37**	**21/37**	**14/60**	**22/40**	**18/63**	**22/37**	19/58	16/35	5/7				
	**23%**	**35%**	**56%**	**23%**	**55%**	**29%**	**59%**	33%	46%	71%				
BCL2	18/25	25/36	29/37	9/26	27/72	43/61	29/37	37/56	30/35	5/7				
	72%	69%	78%	35%	38%	70%	78%	66%	86%	71%				
CyD1	9/18	9/28	16/34	21/46	12/34	21/48	13/32	17/45	15/30	2/5				
	50%	32%	47%	46%	35%	44%	41%	38%	50%	40%				

**Figure 2:**
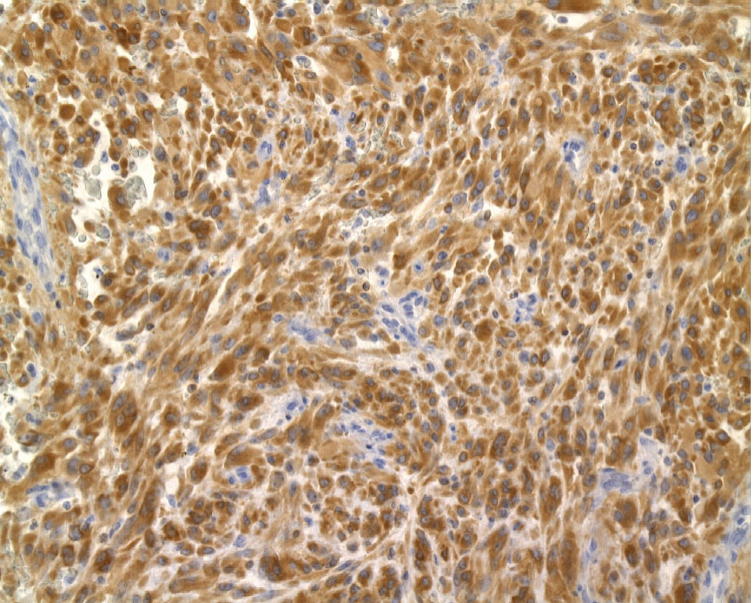
Strong cytoplasmic BCL-2 expression in a GIST from the large intestine showing spindle cell differentiation.

CyD1: Cyclin D1, Figures 2/10 describe 2 out of 10 cases, **bold**: statistical significance, HPF: microscopic high power fields, **F**: Fletcher [10], **M **Miettinen [11], prob malign: probably malignant

**Table 3:** Correlation of P53, BCL-2 and Cyclin D1 expression in KIT positive GISTs with clinicopathological features

	**P53**	**BCL-2**	**Cyclin D1**
Tumor size	**0.01**	0.408	0.243
Mitotic rate	**0.013**	0.207	0.625
Risk of malignancy (Fletcher [10])	**<0.0001**	0.36	0.776
Risk of malignancy (Miettinen [11])	**0.001**	0.196	0.512
Nuclear atypia	**0.02**	0.676	0.38
Proliferative activity (Ki67)	**0.01**	0.794	0.352
Histological subtype	**0.002**	0.391	0.782
Tumor site	0.099	0.118	0.573

**Bold**: significant probability

With regard to epithelioid differentiation, 22 GISTs were P53 positive, of which 12 were localized in the stomach and 10 in the intestine.

Primary resections from GISTs with metastases showed P53 label more often than those without (65% vs. 39%). Survival analysis showed P53 positive tumors in 70% (7/10) of the patients who died, but only in 18% (7/39) survivals. The outcome of patients with P53 positive GISTs tended to be poorer than in negative cases, but these data were not significant.

There was neither a correlation between P53 and BCL-2 expression nor between P53 and Cyclin D1 expression.

Diffuse cytoplasmic BCL-2 staining was found in 69% (72/104) of the GISTs; it was of moderate intensity in 26 cases and of strong intensity in 54 cases (Fig. 2). This staining pattern was very similar to that of CD34, which was found in 73% (76/104) of the GISTs. Almost half of the tumors (48%) coexpressed BCL-2 and CD34 (p = 0.002). All CD34 negative GISTs were positive for BCL-2, and all BCL-2 negatives were positive for CD34. No GIST was negative for both, CD34 and BCL-2. We observed BCL-2 predominantly in GISTs of the small intestine (86%), whereas CD34 labeled significantly gastric GISTs (91%; p = 0.001). The correlation between BCL-2 expression, tumor size and mitotic rate was not significant (Tables 2 and 3).

**Figure 3:**
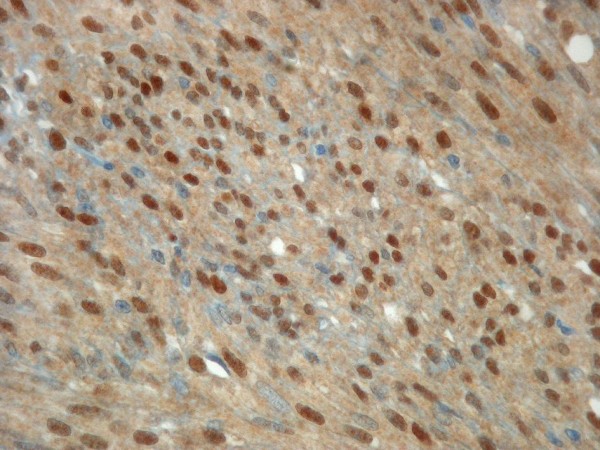
Nuclear Cyclin D1 immunoreactivity in a metastasized gastric GIST, 6 cm in diameter, showing mixed epithelioid-spindle cell differentiation.

Nuclear Cyclin D1 expression was found in 48% (38/80) of the GISTs (Fig. 3). In 18 of them, more than 30% of the tumor cells were labeled. According to our statistical analyses, Cyclin D1 expression was unrelated to all prognostic parameters as well as to histological subtype and tumor site (Tables 2 and 3). Interestingly, Cyclin D1 expression was detected in only 19% (3/16) of the GISTs with metastases (stomach 1, duodenum 2), but in 62% (8/13) of GISTs without metastases from various tumor sites (stomach 3, duodenum 3, ileum 1, colon 1).

## Discussion

Activating mutations of the genes *c-kit *and *PDGFRα *characterize the tumor entity GIST. The mutation status is important for prognosis and a predictive factor for the response to therapy with the tyrosine kinase receptor inhibitor imatinib (Glivec ^®^) [3,12,34-37]. The risk of malignancy is correlated with tumor size, proliferative activity and DNA ploidy (in preparation) [38,39]. It is conceivable that alterations in the genes involved in cell cycle control may modulate the biological behavior of GIST [15].

We tested a number of gene products on GISTs and found that P53 correlated significantly with large tumor size, high mitotic rate, proliferative activity, Fletcher's and Miettinen's high risk criteria, severe nuclear atypia and with epithelioid differentiation. However, BCL-2 and Cyclin D1 labels did not correlate with the said clinicopathological parameters.

Our data, showing P53 expression significantly associated with established prognostic criteria, were in agreement with most previous GIST studies [16,18,40,41]. P53 expression appeared more often in metastasized GISTs than in tumors with disease-free follow-up. These data indicate the impact of the tumor suppressor gene *p53 *on GIST progression. To our surprise, P53 expression was significantly associated with predominantly epithelioid tumor differentiation, independently of tumor site. In general, gastric GISTs with epithelioid differentiation are characterized by PDGFRα mutations and good prognosis. But high risk has been reported upon epithelioid and mixed cell type GISTs without mutated genes *PDGFRa *and *c-kit *[42]. Such tumors develop at different sites. Since epithelioid GISTs have been described with pleomorphic patterns [9], we suggest that our P53 immunoassay detected the epithelioid subtype with variable differentiation in stomach and intestine.

Previous studies have claimed BCL-2 being of diagnostic and prognostic relevance [17,19,21]. Other groups did not attribute prognostic value to BCL-2 [20,29,40]. Our results did not recommend BCL-2 for diagnosis, because it was expressed only in 73% of the GISTs. Otherwise, this percentage does not aid risk assessment, because it was not significant.

However, BCL-2 label was predominantly observed in intestinal GISTs and CD34 expression in gastric GISTs [32]. These findings suggest that GISTs may develop from different somatic subtypes of stem cells, depending on their localization. This hypothesis has received support from two studies showing *PDGFRα *mutations to occur in gastric GISTs and epithelioid differentiation [42,43]. Haller et al. [44] postulated different regulatory mechanisms for the downstream signaling pathway in GISTs with *c-kit *and *PDGFRα *mutations, respectively. Steinert and colleagues [23] found BCL-2 expression frequently associated with progression-free survival of GIST patients who had been treated with imatinib. The authors speculated that the KIT signaling pathway drives GIST development and may induce BCL-2 expression.

Tumors, e.g. mantle cell lymphomas, in which *cyclin D1 *is involved as an oncogene, overexpression or aberrant expression has been supposed to correlate with poor prognosis [45]. In contrast, the outcome was worse for patients when Cyclin D1 expression was low in their GISTs [29]. We found reduced Cyclin D1 expression in GISTs with metastases. However, the survival records were too sparse for statistic analysis. Finally, Cyclin D1 expression did not correlate with the important prognostic indicators like tumor size and mitotic rate. We are therefore unable to confirm that reduced levels of Cyclin D1 represent an unequivocal prognostic factor in GISTs.

## Conclusion

Our investigation confirmed P53 being a powerful immunohistochemical marker for predicting the risk of malignancy in GISTs. Alterations in this tumor suppressor gene seemingly increase the risk of malignancy during tumor progression. In addition, we found P53 significantly correlated with predominantly epithelioid differentiation, independent of tumor site. The antiapoptotic protein BCL-2 and the cell cycle regulator Cyclin D1, however, appeared not helpful in GIST diagnosis and prognosis.

## Competing interests

The authors declare that they have no competing interests.

## Authors' contributions

UP carried out the immunoassays, made one independent report on the immunohistochemical data, performed the statistical analysis and drafted the manuscript. NSG made the histological slides and another independent immunohistochemical report. This manuscript is part of her M.D. thesis. GK participated in the design of the study and its coordination. HM and ACF contributed important intellectual content to complete the project. All authors read and approved the final manuscript.

## Pre-publication history

The pre-publication history for this paper can be accessed here:



## References

[B1] Hornick JL, Fletcher CD (2007). The role of KIT in the management of patients with gastrointestinal stromal tumors. Hum Pathol.

[B2] Steigen SE, Eide TJ, Wasag B, Lasota J, Miettinen M (2007). Mutations in gastrointestinal stromal tumors – a population-based study from Northern Norway. APMIS.

[B3] Lasota J, Miettinen M (2008). Clinical significance of oncogenic KIT and PDGFRA mutations in gastrointestinal stromal tumours. Histopathology.

[B4] Hirota S, Isozaki K, Moriyama Y, Hashimoto K, Nishida T, Ishiguro S, Kawano K, Hanada M, Kurata A, Takeda M, Muhammad TG, Matsuzawa Y, Kanakura Y, Shinomura Y, Kitamura Y (1998). Gain-of-function mutations of c-kit in human gastrointestinal stromal tumors. Science.

[B5] Hirota S, Nishida T, Isozaki K, Taniguchi M, Nakamura J, Okazaki T, Kitamura Y (2001). Gain-of-function mutation at the extracellular domain of KIT in gastrointestinal stromal tumours. J Pathol.

[B6] Taniguchi M, Nishida T, Hirota S, Isozaki K, Ito T, Nomura T, Matsuda H, Kitamura Y (1999). Effect of c-kit mutation on prognosis of gastrointestinal stromal tumors. Cancer Res.

[B7] Heinrich MC, Corless CL, Duensing A, McGreevey L, Chen CJ, Joseph N, Singer S, Griffith DJ, Haley A, Town A, Demetri GD, Fletcher CD, Fletcher JA (2003). PDGFRA activating mutations in gastrointestinal stromal tumors. Science.

[B8] Weiss SW, Goldblum JR (2001). Soft tissue tumors.

[B9] Reichardt P, Hohenberger P (2006). Gastrointestinale Stromatumoren (GIST).

[B10] Fletcher CD, Berman JJ, Corless C, Gorstein F, Lasota J, Longley BJ, Miettinen M, O'Leary TJ, Remotti H, Rubin BP, Shmookler B, Sobin LH, Weiss SW (2002). Diagnosis of gastrointestinal stromal tumors: A consensus approach. Hum Pathol.

[B11] Miettinen M, El Rifai W, Sobin HL, Lasota J (2002). Evaluation of malignancy and prognosis of gastrointestinal stromal tumors: a review. Hum Pathol.

[B12] Wardelmann E, Neidt I, Bierhoff E, Speidel N, Manegold C, Fischer HP, Pfeifer U, Pietsch T (2002). c-kit mutations in gastrointestinal stromal tumors occur preferentially in the spindle rather than in the epithelioid cell variant. Mod Pathol.

[B13] Antonescu CR, Viale A, Sarran L, Tschernyavsky SJ, Gonen M, Segal NH, Maki RG, Socci ND, DeMatteo RP, Besmer P (2004). Gene expression in gastrointestinal stromal tumors is distinguished by KIT genotype and anatomic site. Clin Cancer Res.

[B14] DeMatteo RP, Gold JS, Saran L, Gonen M, Liau KH, Maki RG, Singer S, Besmer P, Brennan MF, Antonescu CR (2008). Tumor mitotic rate, size, and location independently predict recurrence after resection of primary gastrointestinal stromal tumor (GIST). Cancer.

[B15] Gunawan B, Bergmann F, Hoer J, Langer C, Schumpelick V, Becker H, Fuzesi L (2002). Biological and clinical significance of cytogenetic abnormalities in low-risk and high-risk gastrointestinal stromal tumors. Hum Pathol.

[B16] Hillemanns M, Pasold S, Bottcher K, Höfler H (1998). Prognostic factors of gastrointestinal stromal tumors of the stomach. Verh Dtsch Ges Pathol.

[B17] Panizo-Santos A, Sola I, Vega F, de Alava E, Lozano MD, Idoate MA, Pardo-Mindan J (2000). Predicting metastatic risk of gastrointestinal stromal tumors: role of cell proliferation and cell cycle regulatory proteins. Int J Surg Pathol.

[B18] Al Bozom IA (2001). p53 expression in gastrointestinal stromal tumors. Pathol Int.

[B19] Cunningham RE, Abbondanzo SL, Chu WS, Emory TS, Sobin LH, O'Leary TJ (2001). Apoptosis, bcl-2 expression, and p53 expression in gastrointestinal stromal/smooth muscle tumors. Appl Immunohistochem Mol Morphol.

[B20] Sabah M, Cummins R, Leader M, Kay E (2006). Altered expression of cell cycle regulatory proteins in gastrointestinal stromal tumors: markers with potential prognostic implications. Hum Pathol.

[B21] Suster S, Fisher C, Moran CA (1998). Expression of bcl-2 oncoprotein in benign and malignant spindle cell tumors of soft tissue, skin, serosal surfaces, and gastrointestinal tract. Am J Surg Pathol.

[B22] Noguchi T, Sato T, Takeno S, Uchida Y, Kashima K, Yokoyama S, Muller W (2002). Biological analysis of gastrointestinal stromal tumors. Oncol Rep.

[B23] Steinert DM, Oyarzo M, Wang X, Choi H, Thall PF, Medeiros LJ, Raymond AK, Benjamin RS, Zhang W, Trent JC (2006). Expression of Bcl-2 in gastrointestinal stromal tumors: correlation with progression-free survival in 81 patients treated with imatinib mesylate. Cancer.

[B24] Sherr CJ (1996). Cancer cell cycles. Science.

[B25] Alao JP (2007). The regulation of cyclin D1 degradation: roles in cancer development and the potential for therapeutic invention. Mol Cancer.

[B26] Hunter T, Pines J (1991). Cyclins and cancer. Cell.

[B27] Michalides RJ (1999). Cell cycle regulators: mechanisms and their role in aetiology, prognosis, and treatment of cancer. J Clin Pathol.

[B28] Gillett C, Smith P, Gregory W, Richards M, Millis R, Peters G, Barnes D (1996). Cyclin D1 and prognosis in human breast cancer. Int J Cancer.

[B29] Wong NA, Young R, Malcomson RD, Nayar AG, Jamieson LA, Save VE, Carey FA, Brewster DH, Han C, Al Nafussi A (2003). Prognostic indicators for gastrointestinal stromal tumours: a clinicopathological and immunohistochemical study of 108 resected cases of the stomach. Histopathology.

[B30] Nakamura N, Yamamoto H, Yao T, Oda Y, Nishiyama K, Imamura M, Yamada T, Nawata H, Tsuneyoshi M (2005). Prognostic significance of expressions of cell-cycle regulatory proteins in gastrointestinal stromal tumor and the relevance of the risk grade. Hum Pathol.

[B31] Miettinen M, Lasota J (2006). Gastrointestinal stromal tumors: pathology and prognosis at different sites. Semin Diagn Pathol.

[B32] Rudolph P, Chiaravalli AM, Pauser U, Oschlies I, Hillemanns M, Gobbo M, Marichal M, Eusebi V, Hofler H, Capella C, Kloppel G (2002). Gastrointestinal mesenchymal tumors – immunophenotypic classification and survival analysis. Virchows Arch.

[B33] Hall PA, Ray A, Lemoine NR, Midgley CA, Krausz T, Lane DP (1991). p53 immunostaining as a marker of malignant disease in diagnostic cytopathology. Lancet.

[B34] Rubin BP, Singer S, Tsao C, Duensing A, Lux ML, Ruiz R, Hibbard MK, Chen CJ, Xiao S, Tuveson DA, Demetri GD, Fletcher CD, Fletcher JA (2001). KIT activation is a ubiquitous feature of gastrointestinal stromal tumors. Cancer Res.

[B35] Corless CL, McGreevey L, Haley A, Town A, Heinrich MC (2002). KIT mutations are common in incidental gastrointestinal stromal tumors one centimeter or less in size. Am J Pathol.

[B36] Lasota J, Dansonka-Mieszkowska A, Sobin LH, Miettinen M (2004). A great majority of GISTs with PDGFRA mutations represent gastric tumors of low or no malignant potential. Lab Invest.

[B37] Wardelmann E, Buttner R, Merkelbach-Bruse S, Schildhaus HU (2007). Mutation analysis of gastrointestinal stromal tumors: increasing significance for risk assessment and effective targeted therapy. Virchows Arch.

[B38] Rudolph P, Gloeckner K, Parwaresch R, Harms D, Schmidt D (1998). Immunophenotype, proliferation, DNA ploidy, and biological behavior of gastrointestinal stromal tumors: a multivariate clinicopathologic study. Hum Pathol.

[B39] Emory TS, Sobin LH, Lukes L, Lee DH, O'Leary TJ (1999). Prognosis of gastrointestinal smooth-muscle (stromal) tumors: dependence on anatomic site. Am J Surg Pathol.

[B40] Feakins RM (2005). The expression of p53 and bcl-2 in gastrointestinal stromal tumours is associated with anatomical site, and p53 expression is associated with grade and clinical outcome. Histopathology.

[B41] Sabah M, Cummins R, Leader M, Kay E (2006). Altered expression of cell cycle regulatory proteins in gastrointestinal stromal tumors: markers with potential prognostic implications. Hum Pathol.

[B42] Wardelmann E, Hrychyk A, Merkelbach-Bruse S, Pauls K, Goldstein J, Hohenberger P, Losen I, Manegold C, Buttner R, Pietsch T (2004). Association of platelet-derived growth factor receptor alpha mutations with gastric primary site and epithelioid or mixed cell morphology in gastrointestinal stromal tumors. J Mol Diagn.

[B43] Lasota J, Dansonka-Mieszkowska A, Sobin LH, Miettinen M (2004). A great majority of GISTs with PDGFRA mutations represent gastric tumors of low or no malignant potential. Lab Invest.

[B44] Haller F, Gunawan B, von Heydebreck A, Schwager S, Schulten HJ, Wolf-Salgo J, Langer C, Ramadori G, Sultmann H, Fuzesi L (2005). Prognostic role of E2F1 and members of the CDKN2A network in gastrointestinal stromal tumors. Clin Cancer Res.

[B45] Quintanilla-Martinez L, Davies-Hill T, Fend F, Calzada-Wack J, Sorbara L, Campo E, Jaffe ES, Raffeld M (2003). Sequestration of p27Kip1 protein by cyclin D1 in typical and blastic variants of mantle cell lymphoma (MCL): implications for pathogenesis. Blood.

